# Mindfulness techniques for athletic excellence: the mediating role of mental resilience and moderating effect of emotional intelligence

**DOI:** 10.3389/fpsyg.2025.1556619

**Published:** 2025-06-25

**Authors:** Qi Qi

**Affiliations:** School of Physical Education, Xinxiang Medical University, Xinxiang, China

**Keywords:** mindfulness techniques, awareness, non-judgmental acceptance, focused attention, mental resilience, emotional intelligence, athletic performance

## Abstract

**Introduction:**

The study anchors on the conservation of resource theory and examines the impact of mindfulness techniques on athletic performance through the mediating role of mental resilience and the moderating effect of emotional intelligence.

**Methods:**

Data were collected from 332 athletes in China using a time-lagged survey method over three waves. The proposed model was tested using partial least squares structural equation modeling (PLS-SEM).

**Results:**

The results demonstrate that awareness, non-judgmental acceptance, and focused attention, as dimensions of mindfulness, significantly enhance mental resilience. In turn, mental resilience positively influences athletic performance. Additionally, emotional intelligence moderates the relationship between mindfulness techniques and mental resilience, as well as between mindfulness techniques and athletic performance. The mediation analysis confirms that mental resilience partially mediates the relationship between mindfulness and athletic performance. The goodness-of-fit index (GFI) confirms the adequacy of the model.

**Discussion:**

These findings contribute to the understanding of how mindfulness and emotional intelligence interplay to foster mental resilience and enhance athletic outcomes. Practical implications for coaches and sports psychologists are discussed from the perspective of physical education, suggesting that targeted mindfulness and emotional intelligence interventions may improve athletes’ performance and well-being.

## Introduction

Mindfulness techniques, including focused attention, awareness, and non-judgmental acceptance, have attracted growing interest for their well-documented impact on both psychological and physical functioning (Brenner, 2024; Nien et al., 2023). In sports settings, their relevance lies in their potential to enhance athletic performance—primarily through mechanisms such as improved concentration, emotional regulation, and stress reduction (Rogowska and Tataruch, 2024). Studies consistently report that mindfulness helps athletes reduce anxiety, sharpen focus, and recover from setbacks (Filipe et al., 2021; Jekauc et al., 2024; Mojtahe et al., 2023). However, despite these benefits, the translation of mindfulness into long-term athletic performance improvements remains insufficiently understood.

Specifically, the psychological processes that underlie this relationship—such as mental resilience—have not been thoroughly investigated, creating a critical gap in the literature. While prior research suggests that mindfulness promotes resilience by helping athletes stay composed and recover from adversity (Gameiro et al., 2023), empirical testing of this mediating pathway is limited. This study addresses that limitation by investigating whether mental resilience—defined as the ability to adapt and recover from stress (Chue and Cheung, 2023; Zhan et al., 2024)—acts as a key psychological bridge through which mindfulness techniques affect athletic performance. In high-performance environments, where stress and failure are routine, such psychological fortitude is essential for success.

In addition to this mediating pathway, the current study also explores the moderating role of emotional intelligence—a trait defined by the ability to perceive, understand, and regulate emotions (Mayer et al., 2011). Athletes with higher emotional intelligence may more effectively apply mindfulness techniques to manage stress and sustain focus (Rubio et al., 2022), potentially magnifying their performance outcomes. Despite this theoretical relevance, little empirical work has examined the interactive effect of mindfulness and emotional intelligence in athletic contexts.

To ground the investigation, this study draws on the Conservation of Resources (COR) theory (Hobfoll, 1989), which suggests that individuals strive to protect and optimize valuable psychological resources. Mindfulness techniques may function as resource-conserving strategies that help athletes preserve energy, attention, and emotional equilibrium under competitive pressure (Halbesleben et al., 2014). Emotional intelligence may further enhance this resource-conservation process by enabling athletes to regulate emotions more effectively, thereby strengthening resilience and improving performance.

Accordingly, the current study aims to clarify the mechanisms and boundary conditions through which mindfulness techniques improve athletic performance. It proposes that mindfulness enhances performance indirectly via mental resilience and that this effect is strengthened when emotional intelligence is high. By empirically testing these pathways, the study contributes to both theory and practice in sport psychology.

### Review of literature and conceptual model

This study is grounded in the COR theory (Hobfoll, 1989), which posits that individuals strive to acquire, retain, and protect valuable resources, particularly in high-stress environments such as competitive sports. Psychological resources such as mental resilience and emotional stability are central to athletes’ performance under pressure (Halbesleben et al., 2014). Mindfulness techniques (Harita et al., 2022), by promoting attentional control and emotional regulation, may function as resource-conserving strategies. Likewise, EI (Goleman, 2021; Wong and Law, 2002) may enhance athletes’ ability to utilize mindfulness more effectively, thereby safeguarding these psychological resources and improving athletic outcomes.

While mindfulness (Harita et al., 2022; Jekauc et al., 2024; Mojtahe et al., 2023) and emotional intelligence (Nabilpour et al., 2020; Rodriguez-Romo et al., 2021) have individually been studied in sport psychology, few studies have examined their interactive effects, particularly in relation to athletic performance. Moreover, the role of mental resilience as a psychological mechanism through which mindfulness affects performance has not been adequately tested in empirical research.

The current study addresses this gap by proposing and testing an integrated moderated mediation model in which mindfulness techniques influence athletic performance indirectly through mental resilience and conditionally through EI. This approach adds theoretical and applied value by offering a holistic view of how psychological training can support performance. Additionally, the inclusion of EI in this framework supports the development of standardized interventions that account for individual differences in emotional capacities.

Accordingly, this research aims to:

(1) *Examine the influence of mindfulness techniques (awareness, non-judgmental acceptance, and focused attention) on athletic performance through the mediating role of mental resilience, and*
(2) *Explore the moderating effect of emotional intelligence on the relationship between mindfulness and both mental resilience and performance.*

**Figure 1:**
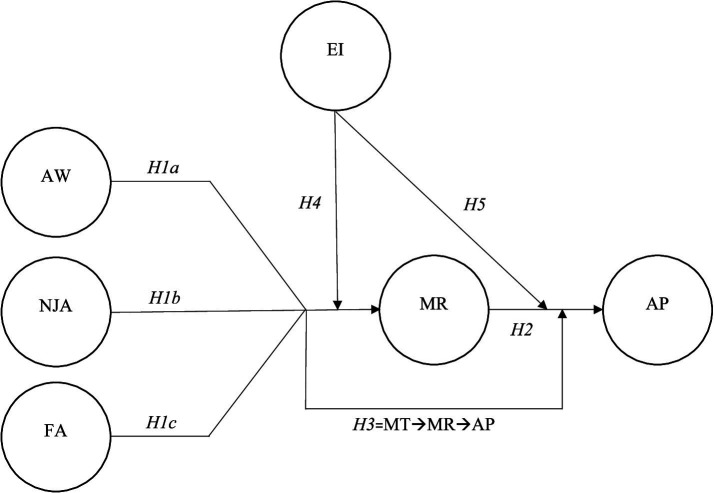
Conceptual model. MT, mindfulness techniques; AW, awareness; NJA, non-judgmental acceptance; FA, focused attention; MR, mental resilience; EI, emotional intelligence; AP, athletic performance.

The study’s conceptual model is posited as follows (Figure 1):

### Hypotheses development

#### Mindfulness techniques and mental resilience

Mindfulness techniques, commonly grouped into practices such as focused attention, non-judgmental acceptance, and awareness (Brenner, 2024; Nien et al., 2023), have been widely studied for their role in promoting psychological well-being. Focused attention involves concentrating on a single object, thought, or sensation, such as breathing, and serves as a key tool in redirecting attention away from stressful stimuli (Anālayo, 2019). By strengthening the athlete’s ability to focus on the present moment, focused attention enhances their capacity to handle distractions and maintain mental clarity during high-pressure situations (Sumantry and Stewart, 2021). Similarly, non-judgmental acceptance allows individuals to observe thoughts and emotions without reacting or assigning value to them, thus helping athletes manage negative emotions, setbacks, or fears of failure (Goisbault et al., 2022). Through this practice, athletes are less likely to be overwhelmed by negative thoughts, fostering a more resilient mindset that can bounce back from adversity (Yau et al., 2021). Awareness, the third component of mindfulness, refers to the conscious perception of one’s body, mind, and environment (Aránega et al., 2020). It helps athletes maintain a holistic understanding of their state during training or competition, allowing for real-time adjustments to their physical or emotional state (Sumantry and Stewart, 2021).

Taken together, these mindfulness techniques function as a holistic practice that can significantly enhance an athlete’s mental resilience. Mental resilience is defined as the capacity to recover quickly from difficulties, adapt to stress, and persist in the face of challenges (Chue and Cheung, 2023). Research suggests that mindfulness can increase resilience by reducing emotional reactivity and fostering a sense of control over one’s responses to stress (Feldman et al., 2007). For instance, Neumann and Tillott (2022) found that individuals who regularly practice mindfulness are better equipped to manage stress and maintain emotional stability, which directly supports resilience. In the athletic context, resilience is particularly important as athletes face a range of stressors, from physical fatigue to the psychological pressure of competition. Mindfulness techniques help athletes remain composed and focused during challenging moments, which in turn strengthens their resilience to bounce back from poor performance or setbacks (Nien et al., 2023).

Given this theoretical framework and the empirical evidence linking mindfulness to improved emotional regulation and stress management, it is hypothesized that:

*H1*: Mindfulness techniques: (a) awareness; (b) non-judgmental acceptance; (c) focused attention, are positively related to mental resilience in athletes.

#### Mental resilience and athletic performance

In sports, athletes are constantly subjected to physical, emotional, and psychological stressors (Jekauc et al., 2024). Mental resilience allows them to cope with these demands, enabling consistent performance, even under adverse conditions. For example, mentally resilient athletes can maintain focus and motivation during periods of intense pressure, such as in high-stakes competitions (Zhan et al., 2024). Athletes who possess higher levels of resilience are better equipped to handle failure, recover from injuries, and manage the stress of competition, which ultimately enhances their overall performance (Connor and Davidson, 2003).

Research consistently demonstrates that mental resilience is a crucial determinant of athletic success. Studies have shown that athletes with higher resilience not only perform better in competitive environments but also display greater perseverance and adaptability during setbacks (Marheni et al., 2024). For instance, athletes who can maintain their composure during competitive stress or bounce back quickly from a poor performance demonstrate the positive impact of resilience on their athletic outcomes (Blanco-García et al., 2021; Gupta and McCarthy, 2022). These findings suggest that resilience acts as a buffer, allowing athletes to manage the demands of training and competition more effectively, leading to improved performance outcomes. Thus, mental resilience plays an essential role in sustaining high levels of athletic performance across varied conditions.

Moreover, resilience enhances athletes’ ability to engage in deliberate practice, a key factor in improving performance over time. Deliberate practice involves repetitive, purposeful activities aimed at skill improvement (Ericsson, 2020), and mentally resilient athletes are more likely to engage in such practices, despite the challenges they face. Resilient athletes are also more capable of maintaining a positive mindset during training and competition, which has been shown to positively affect performance outcomes (Hambrick et al., 2020). This connection between resilience and deliberate practice underscores the vital role that mental resilience plays in enhancing both short-term and long-term athletic performance.

Based on this theoretical understanding and empirical evidence, it is hypothesized that:

*H2*: Mental resilience is positively related to athletic performance.

#### Mediating role of mental resilience

Mindfulness techniques, i.e., focused attention, awareness, and non-judgmental acceptance, are known for their impact on psychological well-being, particularly in managing stress and emotional regulation (Filipe et al., 2021; Mojtahe et al., 2023). As previously discussed, these techniques help athletes stay present, manage negative emotions, and maintain focus, which can significantly influence their mental resilience. Mental resilience, in turn, is a critical factor in athletic performance, as it enables athletes to recover from adversity, handle competitive pressure, and sustain performance over time (Baker et al., 2020). However, while the direct effects of mindfulness on performance are valuable, the mechanism through which this occurs has not been thoroughly explored. This study proposes that mental resilience acts as a mediator, explaining how mindfulness techniques translate into better athletic performance.

Mindfulness helps athletes manage stress, reduce anxiety, and stay calm under pressure, which are all aspects that contribute to building resilience (Brenner, 2024). Through practices like focused attention and non-judgmental acceptance, athletes can regulate their emotions more effectively, preventing emotional exhaustion and allowing them to maintain psychological stability (Sumantry and Stewart, 2021). As athletes develop greater resilience through mindfulness, they are better equipped to handle setbacks and stressful situations, such as performance failure or competition-related stress (Zhan et al., 2024). This resilience not only helps them cope with immediate challenges but also enhances their ability to perform over time, leading to sustained improvements in athletic performance. Thus, mental resilience becomes the key mechanism through which mindfulness affects performance.

The Conservation of Resources (COR) theory (Hobfoll, 1989) provides a strong theoretical foundation for understanding this mediation process. According to COR theory, individuals strive to protect and conserve their psychological and emotional resources, especially when facing stress (Tang et al., 2022). Mindfulness techniques help athletes conserve their cognitive and emotional resources by improving their ability to focus and manage emotions. Mental resilience, in this context, acts as a resource that allows athletes to navigate stressors and maintain their focus on performance (Blanco-García et al., 2021). Through the cultivation of mindfulness, athletes can build resilience, which in turn helps them allocate their resources toward improving their performance, rather than being depleted by stress or emotional turmoil (Brown and Ryan, 2003).

Moreover, empirical evidence supports the idea that resilience mediates the relationship between mindfulness and performance. Studies have shown that athletes who practice mindfulness not only improve their emotional regulation but also develop the psychological strength needed to persevere through difficult situations (Feldman et al., 2007; Filipe et al., 2021; Goisbault et al., 2022). For example, mindfulness interventions in athletes have been linked to reduced stress and improved mental resilience (Liu et al., 2022), which ultimately contributes to better performance outcomes. Given the robust connection between mindfulness and resilience, and the established role of resilience in promoting athletic success, it is reasonable to hypothesize that resilience mediates the positive effects of mindfulness on performance.

Thus, it is hypothesized that:

*H3*: Mental resilience mediates the relationship between mindfulness techniques: (a) awareness; (b) non-judgmental acceptance; (c) focused attention, and athletic performance.

#### Moderating role of emotional intelligence

Emotional intelligence (EI) is defined as the ability to perceive, understand, regulate, and manage emotions in oneself and others (Cherniss et al., 2006). It involves recognizing emotional signals, both internally and externally, and utilizing this emotional awareness to guide thoughts and actions (Goleman, 2021). EI is critical in managing interpersonal relationships, especially in high-stress environments such as athletics, where emotional regulation can influence performance outcomes. High levels of EI are linked to improved emotional regulation, stress management, and the ability to cope with adversities, making it a relevant construct in the context of athletic performance (Laborde et al., 2016).

Emotional intelligence is generally conceptualized through four dimensions: self-emotion appraisal, others’ emotion appraisal, use of emotion, and regulation of emotion (Wong and Law, 2002).

1. Self-emotion appraisal refers to the ability to recognize and understand one’s own emotions. Athletes with high self-emotion appraisal are more adept at identifying stress or anxiety in themselves and are therefore better able to regulate these emotions during competitions (Wong and Law, 2002).
2. Others’ emotion appraisal involves the recognition of emotions in others. In team sports, athletes with strong skills in this dimension can understand the emotional states of teammates and opponents, allowing them to adjust their behavior or strategies accordingly (Wong and Law, 2002).
3. Use of emotion refers to the ability to harness emotions to facilitate performance. Athletes with this skill can channel emotions, such as excitement or frustration, into motivation and focus, enhancing their competitive edge (Wong and Law, 2002).
4. Regulation of emotion refers to the ability to effectively manage and control emotional responses, preventing emotional overwhelm during high-pressure situations. This skill allows athletes to stay composed and focused, even when faced with setbacks or challenges (Wong and Law, 2002).

Holistically, emotional intelligence enhances an athlete’s ability to manage the emotional complexities of competition. By integrating the four dimensions of EI, athletes are not only more capable of regulating their own emotions but can also respond to the emotional dynamics within a team or competitive setting (Kopp and Jekauc, 2018). For athletes, high EI translates to better emotional regulation under pressure, enhanced communication and cooperation with teammates, and the ability to bounce back from stress or failure. Importantly, EI plays a key role in reducing performance anxiety and improving focus, both of which are critical to success in athletic competitions (Laborde et al., 2018).

In the context of this study, emotional intelligence is proposed to moderate the relationship between mindfulness techniques and mental resilience (and athletic performance). While mindfulness techniques like focused attention and non-judgmental acceptance improve athletes’ ability to stay present and regulate emotions (Mojtahe et al., 2023), the effectiveness of these techniques may depend on the athlete’s level of EI. Athletes with high emotional intelligence are likely to derive greater benefits from mindfulness techniques, as they can better perceive and regulate the emotions that arise during practice and competition. For example, an athlete with high EI might use mindfulness to focus their attention on calming their mind during a stressful competition, whereas an athlete with lower EI may struggle to fully leverage mindfulness techniques in emotionally intense situations (Jiménez-Picón et al., 2021).

The Conservation of Resources (COR) theory (Hobfoll, 1989) further supports this moderating role, as athletes with higher EI are better equipped to conserve their emotional resources during competition. By effectively managing their emotions, these athletes experience less emotional exhaustion–leveraging mental resilience, allowing them to channel their focus and energy into their performance. Thus, emotional intelligence enhances the efficacy of mindfulness practices by maximizing athletes’ emotional resources, leading to enhanced resilience and better performance outcomes (Miao et al., 2018). On the other hand, athletes with lower emotional intelligence may struggle to manage stress, limiting the benefits of mindfulness on performance.

In addition, empirical research supports the role of EI as a moderator. Studies have shown that athletes with higher levels of EI perform better under pressure, largely due to their superior emotional regulation and ability to stay focused during stressful situations (Lane et al., 2009; Rubio et al., 2022; Zamanian et al., 2011). Given that mindfulness techniques are designed to help athletes regulate their emotions and stay present, it is likely that athletes with high EI will experience an amplified positive effect from these techniques. Therefore, emotional intelligence can be seen as a critical boundary condition in the relationship between mindfulness and mental resilience (and athletic performance).

Based on this reasoning, it is hypothesized that:

*H4*: Emotional intelligence moderates the relationship between mindfulness techniques and mental resilience, such that the relationship is stronger for athletes with higher emotional intelligence.

*H5*: Emotional intelligence moderates the relationship between mindfulness techniques and athletic performance, such that the relationship is stronger for athletes with higher emotional intelligence.

## Methods and procedures

### Participants

This study employed a purposive sampling method to select athletes from various sports organizations across China. The inclusion criteria required that participants (1) be currently active in either individual or team sports, (2) have at least 1 year of competitive experience, and (3) be aged 18 or above. Athletes who were injured during the study period or had withdrawn from regular training or competition were excluded.

The sample included athletes from a diverse range of sports, including track and field, swimming, basketball, football, volleyball, tennis, martial arts, and gymnastics. This diversity allowed for the inclusion of both individual and team sports, which may differ in terms of psychological demands such as mindfulness and emotional intelligence. All participants received an information sheet detailing the study’s purpose, their rights, data confidentiality, and the voluntary nature of their participation. Informed consent was obtained from all athletes. A total of 450 questionnaires were initially distributed. After removing incomplete or unmatched responses across the three survey waves, the final sample consisted of 332 athletes.

Demographic characteristics are summarized in Table 1. Among the participants, 60% were male and 40% female, with a mean age of 29 years (SD = 0.47). Regarding competitive classification, 42% were amateur, 35% semi-professional, and 23% professional. In terms of experience, 18% had 1–3 years, 34% had 4–6 years, 28% had 7–10 years, and 20% had more than 10 years. Regarding education, 44% held undergraduate degrees and 56% held postgraduate degrees.

**Table 1:** Validity and reliability for constructs.

Indicators	Loadings	AVE	CR	Cronbach’s alpha
Awareness		0.715	0.923	0.879
AW1	0.817			
AW2	0.898			
AW3	0.888			
AW4	0.824			
AW5	0.799			
Non-judgmental acceptance		0.641	0.818	0.721
NJA1	0.788			
NJA2	0.856			
NJA3	0.738			
NJA4	0.819			
NJA5	0.800			
Focused attention		0.657	0.900	0.877
FA1	0.777			
FA2	0.881			
FA3	0.789			
FA4	0.799			
FA5	0.801			
Mental resilience		0.615	0.881	0.834
MR1	0.769			
MR2	0.824			
MR3	0.678			
MR4	0.829			
MR5	0.813			
Emotional intelligence		0.631	0.897	852
EI1	0.691			
EI2	0.788			
EI3	0.767			
EI4	0.720			
EI5	0.891			
EI6	0.810			
EI7	0.879			
EI8	0.700			
EI9	0.802			
EI10	0.829			
EI11	0.791			
EI12	0.786			
EI13	0.839			
EI14	0.820			
EI15	0.791			
EI16	0.775			
Athletic performance		0.614	0.819	0.772
AP1	0.794			
AP2	0.782			
AP3	0.765			
AP4	0.699			
AP5	0.790			
AP6	0.762			
AP7	0.829			
AP8	0.814			
AP9	0.890			
AP10	0.693			

AVE, average variance extracted; CR, composite reliability.

These demographic characteristics play a role in shaping psychological outcomes. Younger athletes may respond differently to mindfulness training compared to seasoned professionals due to cognitive maturity and exposure to structured psychological routines (Gardner and Moore, 2017). Similarly, emotional intelligence may manifest differently in team dynamics than in individual sport contexts. While demographic variables were not the primary focus of this study, these factors should be explored further to examine whether certain groups benefit more from psychological interventions than others.

### Procedure

Prior to data collection, ethical approval was obtained from the Academic Committee of the School of Physical Education, Xinxiang Medical University. The study used a time-lagged survey design across three waves, with an eight-week interval between each wave. This approach was intended to reduce common method variance and establish stronger causal inferences in the moderated mediation model (Maxwell and Cole, 2007).

At Time 1, participants completed measures related to mindfulness techniques and emotional intelligence. Questionnaires were administered in person by trained research assistants at sports facilities and training centers, using printed copies. Standardized instructions were provided to all participants to ensure consistent understanding of the items. At Time 2 (8 weeks later), the same participants were contacted to complete the mental resilience scale. Of the 382 responses from Time 1, 368 were collected, and 355 were successfully matched using self-generated participant codes. At Time 3,8 weeks after Time 2, the athletic performance scale was administered. Ultimately, 332 full responses were retained for analysis across all three waves. To maintain participant anonymity while matching responses across waves, each athlete generated a unique code using their initials and birth year.

### Measures

All constructs were measured using validated scales adapted to the Chinese context. A five-point Likert scale (1 = strongly disagree, 5 = strongly agree) was employed for all items.

Scoring for each scale followed established practices: total scores were calculated by averaging item responses. For all instruments, higher scores indicated stronger presence of the measured construct (e.g., greater mindfulness, higher resilience). Items with negative phrasing were reverse-coded before computation.

Translation of instruments followed a rigorous double-translation (back-translation) protocol. Initially, bilingual experts translated the scales from English to Chinese. A second independent translator then performed a back-translation into English. Any discrepancies were resolved through expert consensus. The translated versions were reviewed by two experts in sports psychology to ensure contextual relevance. Pilot testing with 35 athletes was conducted to confirm clarity and cultural appropriateness. Internal consistency was re-tested for the translated versions (Cronbach’s *α* ranging from 0.85 to 0.90), confirming the reliability of the adapted instruments.

### Mindfulness techniques

The scale was adapted from three established sources: the Mindful Attention Awareness Scale (Brown and Ryan, 2003), the Five Facet Mindfulness Questionnaire (Baer et al., 2006), and the Cognitive and Affective Mindfulness Scale-Revised (Feldman et al., 2007). The items covered awareness, non-judgmental acceptance, and focused attention. As the study was conducted in China, the items were translated from English to Chinese using a double-translation method. A bilingual expert first translated the scales into Chinese, and another independent translator back-translated them into English. Discrepancies were resolved through consensus. A pilot test with 35 athletes was conducted to ensure cultural appropriateness and language clarity. Internal consistency reliability for the 15-item scale was confirmed (Cronbach’s *α* = 0.85).

### Mental resilience

Mental resilience was measured using the five-item version of the Connor-Davidson Resilience Scale (CD-RISC; Connor and Davidson, 2003). The Chinese version, previously validated in local contexts, was adopted with minor linguistic adjustments. Reliability was reassessed during the pilot phase and confirmed (Cronbach’s *α* = 0.89).

### Emotional intelligence

The EI was assessed using the Wong and Law Emotional Intelligence Scale (WLEIS; Wong and Law, 2002), consisting of 16 items across four sub-dimensions. The Chinese version of the WLEIS, commonly used in sport and management research in China, was employed with no modifications. Reliability testing showed high internal consistency (Cronbach’s *α* = 0.90).

### Athletic performance

Athletic performance was measured using a 10-item adaptation of the Athlete Self-Report of Performance scale (Gould et al., 1993). Given the limitations of self-reported performance measures, the items were supplemented with brief explanatory anchors (e.g., “I consistently perform at a high-level during competitions”). Although objective performance indicators such as rankings or coach assessments were not feasible in this study due to time and access limitations. The self-report scale demonstrated acceptable reliability (Cronbach’s *α* = 0.87).

### Empirical analysis

This research utilized partial least squares structural equation modeling (PLS-SEM) to evaluate the proposed associations (Hair et al., 2017). The choice to employ PLS-SEM was predicated on two primary factors: (1) PLS-SEM emphasizes the maximization of explained variance in dependent variables, rendering it suitable for this study, which aims to predict athletic performance and mental resilience rather than confirm theories (Hair et al., 2017); (2) it facilitates the examination of intricate models, including the moderated mediation relationship among mindfulness, emotional intelligence, and mental resilience. PLS-SEM was especially appropriate for evaluating the contextual factors in which emotional intelligence influences the relationship between mindfulness and athletic performance (Henseler et al., 2015).

Furthermore, PLS-SEM is well-suited for studies involving multiple latent constructs, interaction terms, and complex indirect effects—particularly when traditional parametric assumptions (e.g., normal distribution, large sample sizes) are not fully met. Unlike SPSS, which is more appropriate for simple regression or ANOVA-based analyses, PLS-SEM provides the flexibility needed for prediction-oriented models such as the one employed in this study. The decision to use PLS-SEM over covariance-based SEM or SPSS-based techniques stems from its ability to handle formative and reflective constructs, accommodate smaller sample sizes, and model complex paths without requiring multivariate normality.

The use of a singular method and source for data collection (self-reported measures) may pose a risk to the validity of the findings due to common method variance (CMV) (Podsakoff et al., 2003). Despite the implementation of time-lagged data collection and guarantees of anonymity to alleviate common method variance (CMV), additional statistical measures were required. To mitigate CMV, the researcher performed Harman’s single-factor test (Podsakoff et al., 2003). The test posits that if one factor explains over 50% of the covariance, common method variance (CMV) may be a concern. Employing principal component analysis with a single fixed factor and no rotation, the findings revealed that the predominant factor accounted for 29.8% of the variation, well below the 50% threshold. Consequently, CMV does not constitute a substantial risk to the integrity of this study’s results.

Prior to running the structural model, PLS-SEM assumptions such as collinearity, data normality, and multivariate outliers were tested. VIF scores were below the threshold of 3.3, indicating no multicollinearity issues. No significant multivariate outliers were identified using Mahalanobis distance criteria. Skewness and kurtosis values were also within acceptable bounds, indicating no severe violations of normality. Besides, descriptive statistics including means, standard deviations, and correlations were computed using SPSS and presented in supplementary analysis. Finally, missing data were minimal (<2%) and handled through pairwise deletion.

### Measurement model

Hair et al. (2017) suggested to evaluate the measurement model’s quality using internal consistency, reliability, and validity assessments. Internal consistency was assessed by calculating both Cronbach’s alpha and composite reliability (CR). Table 1 displays the Cronbach’s alpha and CR values for all reflecting constructions. The research validated the internal consistency of the outer model, as all Cronbach’s alpha values surpassed the required level of 0.7 (Nunnally and Bernstein, 1994). The composite reliability scores surpassed the 0.7 criterion, demonstrating strong internal consistency for all structures.

To evaluate convergent validity, the researcher calculated the average variance extracted (AVE) and the outer loadings of the constructs. Table 1 indicates that all AVE values exceeded the minimum acceptable criterion of 0.5 (Henseler et al., 2009). Furthermore, all item loadings exceeded the minimal permissible threshold of 0.6, so reinforcing convergent validity.

### Discriminant validity

To ensure discriminant validity, the author employed both the Fornell-Larcker criterion (Fornell and Larcker, 1981) and the Heterotrait-Monotrait ratio (HTMT) approach (Henseler et al., 2015).

#### Fornell–Larcker criterion

Table 2 presents the results of the Fornell-Larcker criterion. According to this method, discriminant validity is confirmed when the square root of the average variance extracted (AVE) for each construct is greater than its highest correlation with any other construct. As shown in Table 2, the diagonal values, representing the square root of the AVE for each construct, are higher than the off-diagonal correlations, thus confirming discriminant validity across the constructs (Hair et al., 2017).

**Table 2:** Discriminant validity (Fornell–Larcker).

Constructs	AW	NJA	FA	MR	EI	AP
AW	0.845					
NJA	0.272	0.800				
FA	0.559	0.647	0.811			
MR	0.648	0.629	0.523	0.784		
EI	0.751	0.721	0.531	0.711	0.794	
AP	0.532	0.490	0.448	0.609	0.612	0.783

AW, awareness; NJA, non-judgmental acceptance; FA, focused attention; MR, mental resilience; EI, emotional intelligence; AP, athletic performance.

#### Heterotrait–Monotrait ratio (HTMT)

We also assessed discriminant validity using the HTMT ratio, which is a more conservative and robust criterion. HTMT values below 0.85 indicate that discriminant validity has been established (Henseler et al., 2015). As shown in Table 3, all HTMT values are below the threshold, further confirming that the constructs exhibit satisfactory discriminant validity.

**Table 3:** Discriminant validity (HTMT).

Constructs	AW	NJA	FA	MR	EI	AP
AW						
NJA	0.319					
FA	0.621	0.427				
MR	0.682	0.532	0.500			
EI	0.723	0.559	0.610	0.710		
AP	0.348	0.623	0.444	0.190	0.481	

AW, awareness; NJA, non-judgmental acceptance; FA, focused attention; MR, mental resilience; EI, emotional intelligence; AP, athletic performance.

### Structural model

After validating the measurement model, the hypothesized structural relationships were assessed using partial least squares structural equation modeling (PLS-SEM). The bootstrapping procedure (with 5,000 resamples) was used to estimate path coefficients (*β*), *t*-values, and confidence intervals (CI) (Hair et al., 2017). Table 4 reports the results of the structural model, which includes both direct effects and interaction effects.

**Table 4:** Direct and interaction effects.

Hypotheses	*β*	CI (5, 95%)	SE	*t*-value	*p*-value	Decision	*f* ^2^	*R* ^2^	*Q* ^2^
*H1a* AW → MR	0.444**	(0.372, 0.528)	0.048	5.831	0.000	Null rejected	0.222	0.493	0.444
*H1b* NJA → MR	0.329**	(0.248, 0.390)	0.071	6.535	0.000	Null rejected	0.321		
*H1c* FA → MR	0.414**	(0.335, 0.492)	0.050	4.960	0.000	Null rejected	0.214		
*H2* MR → AP	0.528**	(0.447, 0.600)	0.048	8.299	0.000	Null rejected	0.184	0.515	0.234
*H4* MT × EI → MR	0.272**	(0.202, 0.356)	0.040	3.566	0.002	Null rejected	0.291		
*H5* MT × EI → AP	0.385*	(0.313, 0.476)	0.062	1.785	0.078	Null rejected	0.249		

AW, awareness; NJA, non-judgmental acceptance; FA, focused attention; MR, mental resilience; EI, emotional intelligence; AP, athletic performance; *significance *p* < 0.1 (1.65); **significance *p* < 0.05 (1.96).

The results show that awareness has a significant positive effect on mental resilience (*β* = 0.444, *t* = 5.831, *p* = 0.000, *f*^2^ = 0.222), supporting H1a. Likewise, non-judgmental acceptance has a significant positive impact on mental resilience (*β* = 0.329, *t* = 6.535, *p* = 0.000, *f*^2^ = 0.321), supporting H1b. Additionally, focused attention significantly predicts mental resilience (*β* = 0.414, *t* = 4.960, *p* = 0.000, *f*^2^ = 0.214), supporting H1c.

Mental resilience also has a significant positive effect on athletic performance (*β* = 0.528, *t* = 8.299, *p* = 0.000, *f*^2^ = 0.184), providing strong support for H2. The *R*^2^ value of 0.515 suggests that mental resilience accounts for a substantial portion of the variance in athletic performance, while Q^2^ indicates strong predictive relevance (*Q*^2^ = 0.234).

**Figure 2:**
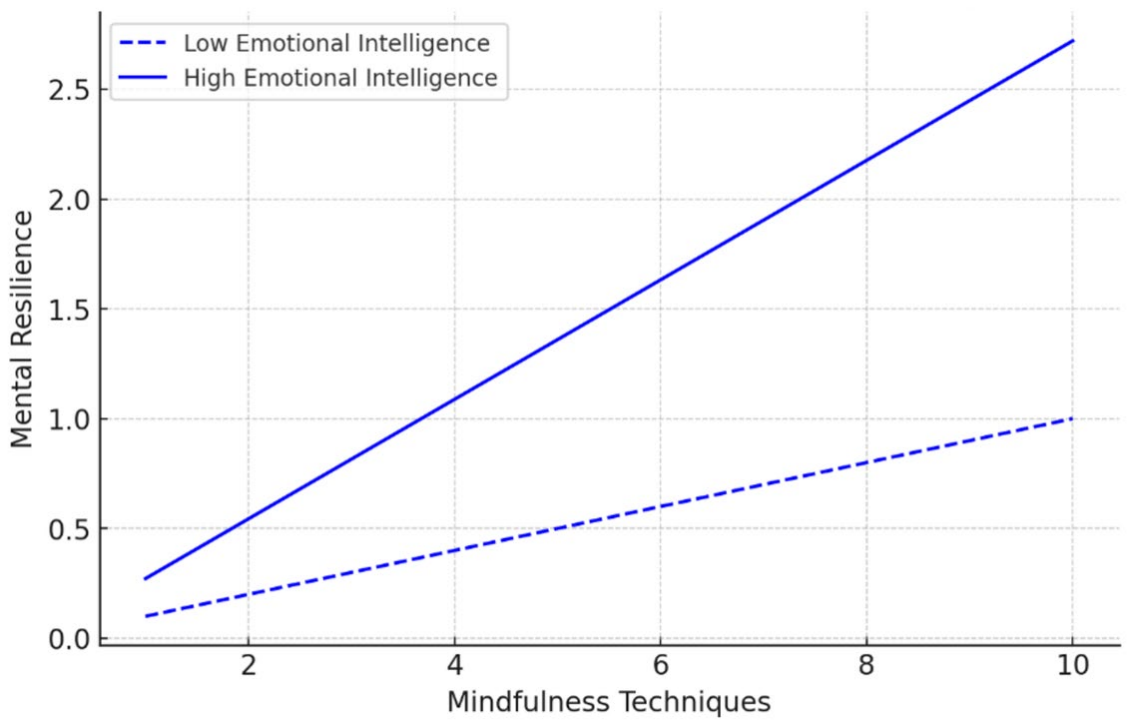
Interaction effect of emotional intelligence on the relationship between mindfulness techniques and mental resilience.

**Figure 3:**
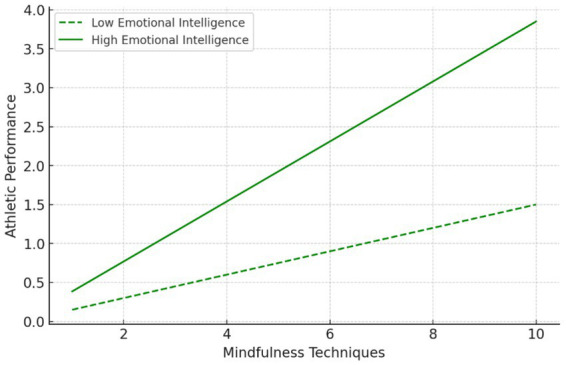
Interaction effect of emotional intelligence on the relationship between mindfulness techniques and athletic performance.

Regarding the interaction effects (Figures 2, 3), the moderation analysis shows that the interaction between mindfulness techniques and emotional intelligence has a significant positive effect on mental resilience (*β* = 0.272, *t* = 3.566, *p* = 0.002, *f*^2^ = 0.291), supporting H4. Furthermore, the interaction of mindfulness techniques and emotional intelligence also significantly influences athletic performance (*β* = 0.385, *t* = 1.785, *p* = 0.078, *f*^2^ = 0.249), supporting H5 at the *p* < 0.1 level. While this effect falls within acceptable thresholds for exploratory research, its statistical significance remains marginal. This is noted as a limitation, and future studies are encouraged to increase sample size or apply Bayesian SEM techniques to improve robustness and power in estimating interaction effects.

To supplement this, interaction plots were generated to visually depict the nature of the moderation effects. Athletes with high emotional intelligence demonstrated stronger positive effects of mindfulness techniques on both mental resilience and athletic performance. These plots are provided in Figures 2, 3.

Furthermore, effect sizes (*f*^2^ = 0.249 for the moderation path to performance) were included to convey the practical relevance of the interactions. Subgroup analysis between amateur and professional athletes is recommended as a future direction, although not conducted due to statistical power concerns.

### Mediation effects

To test the mediation hypotheses, the study followed the analytical approach recommended by Nitzl et al. (2016), utilizing bias-corrected and accelerated (BCa) bootstrapping with 5,000 resamples to generate 95% bootstrap confidence intervals (BCCI) for the indirect effects. The results of the mediation analysis are presented in Table 5.

**Table 5:** Mediation effects.

Effects	Path	*t*-value	95% BCCI		Path	*t*-value	95% BCCI	Decision	VAF
Total effect				Indirect effect					
*H3a* AW → MR	0.462**	8.141	(0.398, 0.538)	AW → MR → AP	0.222**	5.209	(0.167, 0.289)	Supported	48.10%
*H3b* NJA → MR	0.590**	7.290	(0.521, 0.678)	NJA → MR → AP	0.198**	5.632	(0.121, 0.278)	Supported	33.55%
*H3c* FA → MR	0.654**	9.833	(0.578, 0.729)	FA → MR → AP	0.200**	4.235	(0.135, 0.283)	Supported	30.58%

AW, awareness; NJA, non-judgmental acceptance; FA, focused attention; MR, mental resilience; AP, athletic performance; **significance *p* < 0.05 (1.96).

The total effect of awareness on athletic performance, mediated by mental resilience, was significant (*β* = 0.462, *t* = 8.141, 95% BCCI [0.398, 0.538]), confirming the direct relationship. The indirect effect of awareness on athletic performance via mental resilience was also significant (*β* = 0.222, *t* = 5.209, 95% BCCI [0.167, 0.289]), supporting H3a. The variance accounted for (VAF) was 48.10%, indicating partial mediation.

For non-judgmental acceptance, the total effect on athletic performance was significant (*β* = 0.590, *t* = 7.290, 95% BCCI [0.521, 0.678]), and the indirect effect via mental resilience was also significant (*β* = 0.198, *t* = 5.632, 95% BCCI [0.121, 0.278]), supporting H3b. The VAF of 33.55% suggests partial mediation.

Similarly, the total effect of focused attention on athletic performance was significant (*β* = 0.654, *t* = 9.833, 95% BCCI [0.578, 0.729]), and the indirect effect via mental resilience was significant (*β* = 0.200, *t* = 4.235, 95% BCCI [0.135, 0.283]), supporting H3c. The VAF of 30.58% confirms partial mediation. To strengthen the analysis, statistical power for mediation paths was computed using G*Power 3.1. With a sample size of 332, the study achieved a power of 0.92 for detecting medium effect sizes at the 0.05 level. This confirms the adequacy of the sample size for mediation analysis.

### Goodness-of-fit index (GFI)

To assess the overall model fit, the researcher calculated the Goodness-of-Fit Index (GFI), as recommended by Tenenhaus et al. (2005). The GFI is computed as the geometric mean of the average variance extracted (AVE) and the average *R*^2^ of the endogenous constructs. A higher GFI value indicates a better model fit. Table 6 shows the AVE and *R*^2^ values for each construct. The average AVE for the constructs was 0.645, and the average R^2^ was 0.504. The resulting GFI value was 0.571, which exceeds the threshold of 0.36 for a large effect size, indicating that the model demonstrates a good fit (Hoffmann and Birnbrich, 2012). This confirms the model’s overall adequacy in explaining the relationships among the constructs.

**Table 6:** Goodness-of-fit index (GFI).

Constructs	AVE	*R* ^2^
Awareness	0.715	
Non-judgmental acceptance	0.641	
Focused attention	0.657	
Mental resilience	0.615	0.493
Emotional intelligence	0.631	
Athletic performance	0.614	0.515
Average scores	0.645	0.504
*GFI =* $\sqrt{\bar{\text{AVE}}\times \bar{R^{2}}}$	0.571	

AW, awareness; NJA, non-judgmental acceptance; FA, focused attention; MR, mental resilience; EI, emotional intelligence; AP, athletic performance.

## Discussion

This study contributes to the expanding body of literature on mindfulness by exploring its relationship with athletic performance through the mediating role of mental resilience and the moderating role of emotional intelligence. Drawing from the Conservation of Resources (COR) theory (Hobfoll, 1989), the study proposed that mindfulness techniques enhance mental resilience, which in turn improves athletic performance, while emotional intelligence moderates the strength of these effects. The findings largely validate these theoretical projections and offer valuable insights into the mechanisms through which mindfulness impacts athletic outcomes.

The main findings demonstrate that mindfulness techniques—specifically focused attention, awareness, and non-judgmental acceptance—positively influence athletes’ mental resilience, which in turn significantly enhances their athletic performance. Furthermore, emotional intelligence was found to moderate this relationship, strengthening the indirect effect of mindfulness on performance outcomes. These results underscore the dual psychological pathways—resilience and emotion regulation—through which mindfulness exerts its impact in competitive sports contexts.

This research adds to existing studies that have highlighted the positive effects of mindfulness on psychological regulation, particularly in high-pressure environments like sports (Harita et al., 2022; Röthlin et al., 2016). The results indicate that mindfulness techniques—focused attention, awareness, and non-judgmental acceptance—directly contribute to the enhancement of mental resilience, which supports athletes in overcoming stress and adversity. Athletes who engage in mindfulness practices are better equipped to manage the emotional demands of competitive environments, aligning with past research that underscores mindfulness as a critical tool for stress regulation (Dana et al., 2022; Pineau et al., 2014). By extending this understanding, the study demonstrates that mental resilience serves as a crucial mediator between mindfulness and athletic performance, offering a novel insight into how psychological resources are conserved and utilized in athletic contexts. This extends the literature by moving beyond the direct effects of mindfulness, providing evidence that the cultivation of resilience is a key pathway through which mindfulness enhances performance.

Concrete examples in elite sport further validate the theoretical relevance of mental resilience. For instance, athletes competing in sudden-death matches, penalty shootouts, or final rounds of tournaments must manage acute psychological pressure. In such situations, resilient athletes are more likely to rebound quickly from earlier failures, maintain focus, and adapt their strategies (Gucciardi et al., 2017). Resilience enables tennis players to come back after losing a set, basketball players to refocus after missed shots, and gymnasts to regulate fear during high-stakes routines—making it a vital determinant of consistent performance under pressure (Fletcher and Sarkar, 2012; Galli and Gonzalez, 2015).

The mediating role of mental resilience is particularly significant in light of the challenges athletes face in maintaining focus and performance under pressure. Resilience, as the ability to recover from setbacks and adapt to stressful circumstances, is a critical component of athletic success (Fletcher, 2018; Gupta and McCarthy, 2022). The study’s findings suggest that mindfulness techniques bolster this resilience, allowing athletes to maintain composure and focus during competition. Through practices such as focused attention and non-judgmental acceptance, athletes are able to regulate their emotional responses to stress, thereby conserving their psychological resources and enabling them to channel their energy into performance (Vveinhardt and Kaspare, 2022). This insight reinforces the theoretical argument that mental resilience is not only a protective factor in the face of adversity but also a key mediator that explains how mindfulness contributes to sustained athletic performance.

Moreover, this study offers significant contributions to the literature by identifying EI as a moderating factor that shapes the relationship between mindfulness techniques and athletic performance. EI, which encompasses the ability to perceive, understand, regulate, and use emotions effectively, plays a vital role in enhancing athletes’ ability to manage stress and stay focused in competitive settings (Arribas-Galarraga et al., 2019). The results suggest that athletes with higher emotional intelligence derive greater benefits from mindfulness techniques, as their ability to manage and regulate emotions allows them to fully capitalize on the focus and emotional regulation that mindfulness fosters (Ajilchi et al., 2019; Kopp and Jekauc, 2018; Nabilpour et al., 2020). This finding aligns with the COR theory, which posits that individuals who can effectively manage their emotional resources are better positioned to conserve and utilize these resources for improved performance (Hobfoll, 1989). Athletes with high emotional intelligence are thus able to leverage mindfulness techniques more effectively, resulting in enhanced resilience and, ultimately, better performance outcomes.

This contribution is particularly significant in the context of emotion-intensive sports, such as combat sports, team sports, or high-pressure endurance events, where moment-to-moment emotion regulation is pivotal for decision-making and execution. The interaction between EI and mindfulness thus reflects a unique psychological synergy—one that can be harnessed to build not only mental strength but also performance consistency under emotionally taxing conditions.

The moderation of emotional intelligence is particularly insightful as it highlights the role of individual differences in shaping the effectiveness of mindfulness interventions. Previous research has explored emotional intelligence in relation to performance outcomes, noting that athletes with high EI are more likely to regulate their emotions under pressure and maintain focus (Rodriguez-Romo et al., 2021). However, this study is one of the few to empirically test how emotional intelligence interacts with mindfulness techniques, providing evidence that athletes who are emotionally intelligent are better equipped to use mindfulness to enhance their resilience and performance. The moderating effect of EI underscores the importance of developing both mindfulness and emotional intelligence in athletes to optimize their performance, particularly in environments where the ability to manage stress and emotions is critical.

While numerous studies affirm the value of mindfulness in sport, some investigations suggest that its effects are not universally strong or consistent. For example, Ford and Gross (2018) found that mindfulness interventions had small or non-significant effects on some performance metrics in low-pressure environments. Similarly, Noetel et al. (2019) observed that the variability in intervention outcomes may stem from factors such as dosage, delivery mode, or athlete engagement. This study contributes a nuanced perspective by illustrating that the strength of mindfulness effects depends not only on the psychological trait of resilience but also on the moderating presence of emotional intelligence. This layered model enhances theoretical depth by contextualizing mindfulness within broader psychological capacities (Gardner and Moore, 2007).

In combination, these findings provide a comprehensive understanding of the psychological processes that link mindfulness techniques to athletic performance. By demonstrating that mental resilience mediates the relationship between mindfulness and performance, and that emotional intelligence moderates this relationship, the study offers a more nuanced view of how psychological traits and practices interact to influence athletic outcomes. This not only adds depth to the existing literature on mindfulness in sports but also opens new avenues for future research. For instance, future studies could explore interventions designed to simultaneously enhance mindfulness and emotional intelligence, investigating whether such combined interventions lead to even greater improvements in athletic performance.

Furthermore, this study expands the literature by addressing its findings within a specific cultural context—mainland China. While mindfulness originates from Eastern traditions, its integration into contemporary sports psychology within China reflects a resurgence of cultural acceptance, combined with modern evidence-based frameworks. Cultural norms that value harmony, discipline, and self-regulation may synergize with mindfulness practices, influencing how athletes internalize and respond to these interventions (Liu et al., 2022). However, collectivist cultural values may also shape the expression of emotional intelligence differently compared to Western samples, particularly regarding emotional expression or suppression (Goleman, 2021). Recognizing these cultural dimensions adds interpretive depth and calls for future cross-cultural comparisons.

### Limitations and future research

While this study provides important insights into the relationship between mindfulness techniques, mental resilience, and athletic performance, several limitations should be noted. First, the use of self-reported measures for key constructs may introduce bias, such as social desirability, which could affect the accuracy of responses. This concern is particularly relevant for constructs like emotional intelligence and mindfulness, which are susceptible to self-enhancement or recall inaccuracies. Future research could incorporate objective performance metrics or third-party evaluations to enhance the validity of the findings.

Second, the study focuses on a sample of athletes in a specific cultural and competitive context, potentially limiting the generalizability of the results. Future studies should examine diverse athlete populations across various sports and cultural backgrounds to better understand the broader applicability of the findings. Comparative cross-cultural studies, especially between collectivist and individualist societies, may help delineate how cultural norms influence the expression and effectiveness of psychological interventions.

Additionally, while this study identifies mental resilience and emotional intelligence as key psychological factors influencing athletic performance, future research could explore other potential mediators and moderators, such as motivation, team dynamics, or leadership support. Longitudinal studies examining the long-term effects of mindfulness and emotional intelligence on athletic outcomes would also be valuable in determining how these psychological traits evolve over time and their sustained impact on performance. Expanding the scope of research to include interventions designed to simultaneously enhance mindfulness and emotional intelligence may provide more robust insights into how athletes can optimize their psychological resources for peak performance.

### Practical implications

The findings of this study have significant practical implications for athletes, coaches, and sports psychologists aiming to enhance athletic performance through psychological training from the perspective of physical education. First, the positive impact of mindfulness techniques on mental resilience highlights the importance of incorporating mindfulness practices into athletes’ training regimens. Coaches can introduce mindfulness exercises such as focused attention, body scans, and non-judgmental acceptance into daily routines, helping athletes develop greater emotional regulation and resilience under competitive pressure. By cultivating mindfulness, athletes will not only improve their ability to cope with stress but also build the psychological strength needed to recover from setbacks and maintain consistent performance.

Additionally, this study emphasizes the role of emotional intelligence in amplifying the effects of mindfulness on performance. Coaches and sports psychologists should consider assessing and developing athletes’ emotional intelligence alongside mindfulness training. Strategies such as emotional awareness training, reflective practices, and interpersonal skill-building can help athletes better manage their emotions and harness them effectively during competition. Athletes with higher emotional intelligence are better positioned to benefit from mindfulness techniques, allowing them to maintain focus, handle stress, and enhance their performance. Programs that integrate both mindfulness and emotional intelligence training could prove to be especially beneficial in high-pressure environments where emotional regulation is critical to success.

Finally, organizations and sports teams should recognize the value of psychological resources such as mindfulness and emotional intelligence in fostering long-term performance improvement. Investing in mental skills training that targets these areas can enhance athletes’ overall well-being, reduce burnout, and improve team dynamics. As mindfulness helps athletes manage their internal responses to stress, and emotional intelligence aids in managing interpersonal and competitive dynamics, teams that focus on these aspects are likely to see better cohesion and performance. This holistic approach to mental and emotional development not only strengthens individual athletes but also contributes to a more resilient, high-performing team environment.

## Conclusion

The present study highlights the crucial role of mindfulness techniques in enhancing athletic performance through the mediating effect of mental resilience and the moderating role of emotional intelligence from the perspective of physical education. Athletes who develop mindfulness skills are better equipped to manage stress and bounce back from adversity, with emotionally intelligent individuals experiencing even greater benefits. The findings offer practical insights for incorporating mindfulness and emotional intelligence training into athletic development programs. Future research should explore broader contexts and additional psychological factors to further enrich the understanding of how these constructs influence long-term athletic success.

## Data Availability

The raw data supporting the conclusions of this article will be made available by the authors, without undue reservation.
