# Supplementary material for: Mindfulness techniques for athletic excellence: the mediating role of mental resilience and moderating effect of emotional intelligence
Source: Front Psychol. 2025 Jun 25;16:1556619. doi: 10.3389/fpsyg.2025.1556619 (PMC12238952; doi:10.3389/fpsyg.2025.1556619)
Supplement: Supplementary file 1 [file Data_Sheet_1.docx]

**Appendix**

**1. Awareness (a subscale of mindfulness)**

From the **Mindful Attention Awareness Scale (MAAS)** (Brown & Ryan, 2003):

**Items**:

1. I find it difficult to stay focused on what's happening in the present. (R)
2. I do jobs or tasks automatically, without being aware of what I’m doing. (R)
3. I rush through activities without being really attentive to them. (R)
4. I could be experiencing some emotion and not be conscious of it until later. (R)
5. I find myself doing things without paying attention. (R)

**Note**: (R) indicates reverse-coded items.

**2. Non-Judgmental Acceptance (a subscale of mindfulness)**

From the **Five Facet Mindfulness Questionnaire (FFMQ)** (Baer et al., 2006):

**Items**:

1. I criticize myself for having irrational or inappropriate emotions. (R)
2. I tell myself I shouldn’t be feeling the way I’m feeling. (R)
3. I believe some of my thoughts are abnormal or bad, and I shouldn’t think that way. (R)
4. I make judgments about whether my thoughts are good or bad. (R)
5. I think some of my emotions are bad or inappropriate and I shouldn’t feel them. (R)

**3. Focused Attention (a subscale of mindfulness)**

From the **Cognitive and Affective Mindfulness Scale-Revised (CAMS-R)** (Feldman et al., 2007):

**Items**:

1. I can usually describe how I feel at the moment in considerable detail.
2. It is easy for me to keep track of my thoughts and feelings.
3. I am easily distracted. (R)
4. I am able to pay close attention to one thing for a long period of time.
5. I can focus on the present moment, without being distracted by irrelevant thoughts.

**4. Mental Resilience**

From the **Connor-Davidson Resilience Scale (CD-RISC)** (Connor & Davidson, 2003):

**Items**:

1. I am able to adapt when changes occur.
2. I can deal with whatever comes my way.
3. I try to see the humorous side of things when I am faced with problems.
4. I tend to bounce back after illness, injury, or other hardships.
5. I believe I can achieve my goals, even if there are obstacles.

**5. Emotional Intelligence**

From the **Wong and Law Emotional Intelligence Scale (WLEIS)** (Wong & Law, 2002)

**Items:**

**Self-Emotion Appraisal (SEA)**

1. I have a good sense of why I have certain feelings most of the time.
2. I have a good understanding of my own emotions.
3. I really understand what I feel.
4. I always know whether or not I am happy.

**Others' Emotion Appraisal (OEA)**

1. I always know my friends’ emotions from their behavior.
2. I am a good observer of others’ emotions.
3. I am sensitive to the feelings and emotions of others.
4. I have good understanding of the emotions of people around me.

**Use of Emotion (UOE)**

1. I always set goals for myself and then try my best to achieve them.
2. I always tell myself I am a competent person.
3. I am a self-motivated person.
4. I would always encourage myself to try my best.

**Regulation of Emotion (ROE)**

1. I am able to control my temper so that I can handle difficulties rationally.
2. I can always calm down quickly when I am very angry.
3. I have good control of my own emotions.
4. When I am angry, I will try to understand why I am angry.

**6. Athletic Performance**

From the **Athlete Self-Report of Performance** (Gould *et al.,* 1999)

1. I consistently perform at a high level during competitions.
2. I am satisfied with my performance in training.
3. I am able to maintain my energy and effort throughout the entire competition.
4. My overall athletic performance has improved over the past season.
5. I am effective at achieving my performance goals during training.
6. I can perform under pressure in important competitions.
7. I regularly meet or exceed my performance expectations in competitions.
8. My focus and concentration are strong when competing.
9. I perform well even when faced with difficult opponents or conditions.
10. I can recover quickly after poor performance and get back on track during a competition.
